# Sequential Dependencies in Solid and Fluid Intake in Nursing Home Residents with Dementia: A Multistate Model

**DOI:** 10.1093/geroni/igaa057.3362

**Published:** 2020-12-16

**Authors:** Wen Liu, Kristine Williams, Yong Chen

**Affiliations:** 1 University of Iowa, Iowa City, Iowa, United States; 2 University of Kansas, Lawrence, Kansas, United States

## Abstract

Nursing home (NH) residents with dementia commonly experience low food intake leading to negative consequences. While multilevel factors influence intake, evidence is lacking on how intake is sequentially associated. This study examined the temporal association between previous and current solid and fluid intake in NH residents with dementia. We analyzed 160 mealtime videos involving 27 residents and 36 staff (53 dyads) in 9 NHs. The dependent variable was the current intake state (fluid, solid, no-intake). Independent variables included the prior intake state, technique of current intake state (resident-initiated, staff-facilitated), duration between previous and current intakes. Covariates included resident and staff characteristics. Two-way interactions of duration and technique with the prior intake state, and resident comorbidity and dementia severity were examined using Multinomial Logit Models. Interactions were significant for technique by comorbidity, technique by dementia severity, technique by prior fluid and solid intake, and duration by prior fluid intake. Successful previous intake increased odds of current solid and fluid intake. Staff-facilitation (vs. resident-initiation) reduced odds of solid and fluid intake for residents with moderately severe (vs. severe) dementia. Higher morbidity decreased odds of solid intake (vs. no-intake) for staff-facilitated intake. Resident with severe dementia had smaller odds of solid and fluid intake for resident-initiated intake. Longer duration increased odds of transition from liquid to solid intake. Findings supported strong sequential dependencies in intake, indicating the promise of intervening behaviorally to modify transitions to successful intake during mealtime. Findings inform the development and implementation of innovative mealtime assistance programs to promote intake.

